# Development of fully-automated synthesis systems

**DOI:** 10.1155/S1463924694000039

**Published:** 1994

**Authors:** Tohru Sugawara, Shinji Kato, Shigeha Okamoto

**Affiliations:** Molecular Chemistry Laboratory Pharmaceutical Research Division Takeda Chemical Industries Ltd, 17–85, uso Honmachi 2-chome, Todogawa-ku Osaka 532 Japan

## Abstract

This paper describes the development of fully-automated synthesis systems for preparing and isolating various kinds of pharmaceutical compounds. The systems are versatile, and are able to perform most of the chemical reactions currently used in organic chemistry, with the exception of hydrogenation which requires high pressure. An additional benefit is the very user-friendly software.


Journal of Automatic Chemistry, Vol. 16, No. 1 (January–February 1994), pp. 33–42

### Development of fully-automated synthesis systems

**Tohru Sugawara, Shinji Kato and Shigeha Okamoto**
*Molecular Chemistry Laboratory, Pharmaceutical Research Division, Takeda Chemical Industries, Ltd, 17–85, Juso Honmachi 2-chome, Yodogawa-ku, Osaka 532, Japan*

*This paper describes the development of fully-automated synthesis systems for preparing and isolating various kinds of pharmaceutical compounds. The systems are versatile, and are able to perform most of the chemical reactions currently used in organic chemistry, with the exception of hydrogenation which requires high pressure. An additional benefit is the very user-friendly software.*

### Introduction

Pharmaceutical chemists have to carry out extremely routine tasks, such as optimizing reaction conditions and synthesizing many derivatives on the several hundred milligram scale for determining biological activity at the early stage of screening. To release chemists from these time-consuming tasks, the authors have been developing automated synthesis systems equipped with an artificial intelligence (see table 1). With the first system, over 200 derivatives of substituted N-(carboxyalkyl)amino acids [1-4] were synthesized. The design of this first-generation system was based on a one-way operating system from the mixing of reactants to the isolation of products. This paper reports on improved second and third generation systems. They consist of modular units, which can be operated together or independently (see figure 1), and are controlled with user-friendly software.

*Table 1. Automated synthesis systems.*

| Generation | | |
|---|---|---|
| 1st | An unnamed prototype | |
| 2nd | TACOS | Takeda's Automated Computer-Operated System. |
| | MAVIS | Multipurpose Automated Versatile Intelligent System. |
| 3rd | VACOS | Versatile Automated Computer-Operated System. |
| | MATES | Multipurpose Automated Technical Equipment for Synthesis. |
| | TARO | Takeda's Automated Reliable Operation. |
| | EASY | Expert Automated Synthesizer for You. |

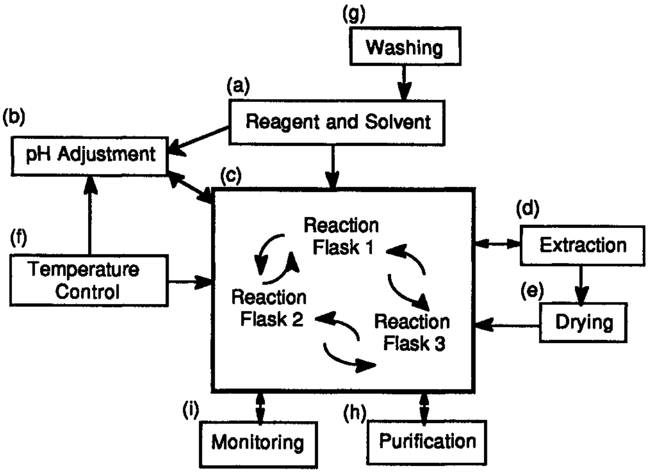

*Figure 1. Schematic diagram of the operating units.*

(*a*) Reagent–solvent supply unit: reservoirs containing all the reagents, solvents and reaction solutions, and individual volumetric tubes fitted with photosensors to allow measurement of the required volumes.
(*b*) pH-adjustment device: flask fitted with a pH electrode to measure the pH of a product solution.
(*c*) Reaction unit: two flasks with thermostatic jackets, condensers and stirrers; one flask with a condenser, stirrer and an oil-bath.
(*d*) Extraction/separation-funnel device: glass funnel fitted with an electric sensor to allow separation of organic and aqueous phases.
(*e*) Drying-tube device: for removing water from organic phases by passing them through a drying agent (for example $Na_2SO_4$).
(*f*) Temperature control unit: a circulation system with hot and cold fluids for the reaction flasks and condensers.
(*g*) Washing-exhaust/drainage unit: wash solvent reservoirs, a diaphragm pump and drainage vessel to enable complete washing of the apparatus after each run.
(*h*) Purification unit: a HPLC device and a fraction collector device.
(*i*) Reaction monitor unit: for real-time sampling and HPLC analysis.

### Design and construction of the hardware

### *General features*

The automated system is composed of a computer control and synthesis components. The latter comprises units for performing various tasks, such as the supply of reagents and solvents, the control of reaction conditions and the purification of products. The latest synthesis system is

0142-0453/94 $10.00 © 1994 Taylor & Francis Ltd.

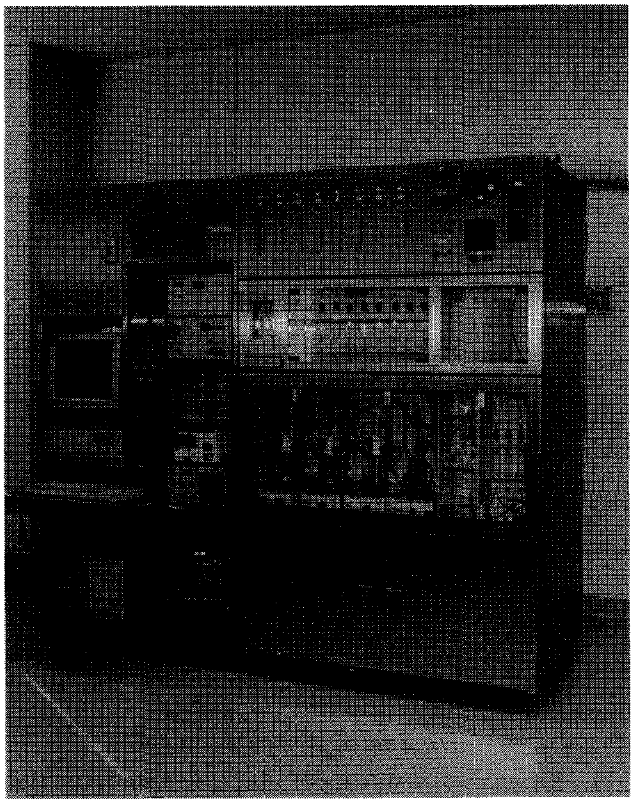

*Figure 2. Photograph of EASY.*

shown in figure 2 and the layouts of the various units of several models are shown in figure 3.

*Synthesis system*

*Reagent and solvent supply unit*

TACOS and MAVIS: The reagent and solvent supply unit (figure 4[*a*]) consists of 16 reservoirs (nine for reactants, RR1–RR9; five for solvents, RS1–RS5; and two for pH adjustment, RS6, RS7); 64 solenoid valves (MTV-21-M6 and MTV-31-M6, Takasago Electric, Inc., Japan); and 12 volumetric tubes (MT1–MT12, seven 10 ml, and five 5 ml), fitted with gas–liquid (G–L) sensors (G–L sensors or photosensors). All the flow lines, which are made of Teflon, are part of a closed system.

Reagent solutions and solvents are introduced into the reservoirs under reduced pressure using a diaphragm pump. The volumes of the reservoirs are 190 ml for solvents, 90 ml for reagents and 150 ml for pH adjustment solutions. The transfer of liquid reagents and solvents to the reaction flasks is performed in two steps. First, the solution is allowed to flow from the reservoir into the volumetric tube. Second, the content of the volumetric tube is transported into the reaction flask immediately after the G–L sensor detects that the tube is full. The user can stock excess amount of reagents and solvents for many runs, as volumes are measured before use. As a result, this generation has mainly become suitable for repetitive syntheses.

*VACOS, MATES, TARO and EASY:* The solvent supply unit (figure 4[*b*]) consists of eight reservoirs (six for solvents, RS1–RS6; and two for pH adjustment, RS7 and RS8) and two measuring tubes (MT1 and MT2). The volumes of the solutions stored in the reagent supply unit, which is made up of nine reservoirs (RR1–RR9), are not measured before transfer to the connected reaction flasks (RF). Therefore it is possible to minimize any loss of the solution in the lines between the reservoirs and measuring tubes—this apparatus is more suitable for reactions that require expensive or unstable reagents.

Apparatus of this type has G–L sensors (PS12–PS14) to check the beginning and the completion of the liquid flow from RR1–RR9. Other features are similar to those listed under TACOS and MAVIS.

*Reaction unit and pH adjustment device*

Figure 5 shows the main reaction unit, of three reaction flasks, and the pH adjusting device. Two reaction flasks, RF1 and RF2, and the pH adjusting flask (PH) have jackets through which the heating/cooling fluid of ethylene glycol is circulated. The reaction temperatures are thermostatically controlled by regulating the flow of either hot or cold fluid. The reaction flask, RF3, is equipped with an oil bath and a regulated heating element so that it can be heated up to 200°C. Its temperature is set freely by keyboard input through an RS232C port.

All these flasks (RF1–RF3, PH) are approximately 100 ml in volume but can be replaced with other sizes of flask. They are equipped with a magnetic or mechanical stirrer, and are connected with each other to allow solutions to be transferred between them. The flow lines are equipped with G–L sensors to check the beginning and the completion of the liquid flow out of the flasks.

Concentration of solutions can be performed in RF1, RF2, and RF3 by heating and bubbling with an inert gas (argon or nitrogen) at the same time. A concentration sensor, which detects the completion of evaporation by tracing the vapour temperature with a thermocouple K(CA), is also attached.

*Extraction-drying unit*

The unit consists of a separation funnel (SF) equipped with a liquid–liquid sensor (L–L sensor, LL), two solution reservoirs (SR0 and SR1), and five drying tubes (DT1–DT5, 50 ml), see figure 6.

The reaction mixtures can be transferred from any of the reaction flasks (RF) to the SF under reduced pressure. Extraction and washing is performed either by stirring vigorously in the RF or by bubbling in the RF or SF. Separation of two immiscible liquid phases, formed after extraction or washing of a reaction mixture, is accomplished by using the L–L sensor to measure the electroconductivity of the two different phases. The lower layer is transferred to either SR0 or SR1. The organic solutions extracted can then be dried by being passed through one of the drying tubes which are prefilled with desiccant such as anhydrous sodium sulphate. To select the drying tube, a position select rotary valve (E1E-023, Uniflows Co., Ltd, Japan) is employed.

The L–L sensor is a simple, but highly sensitive, device which can measure the relative electroconductivity of liquids. The difference between the conductivity in the

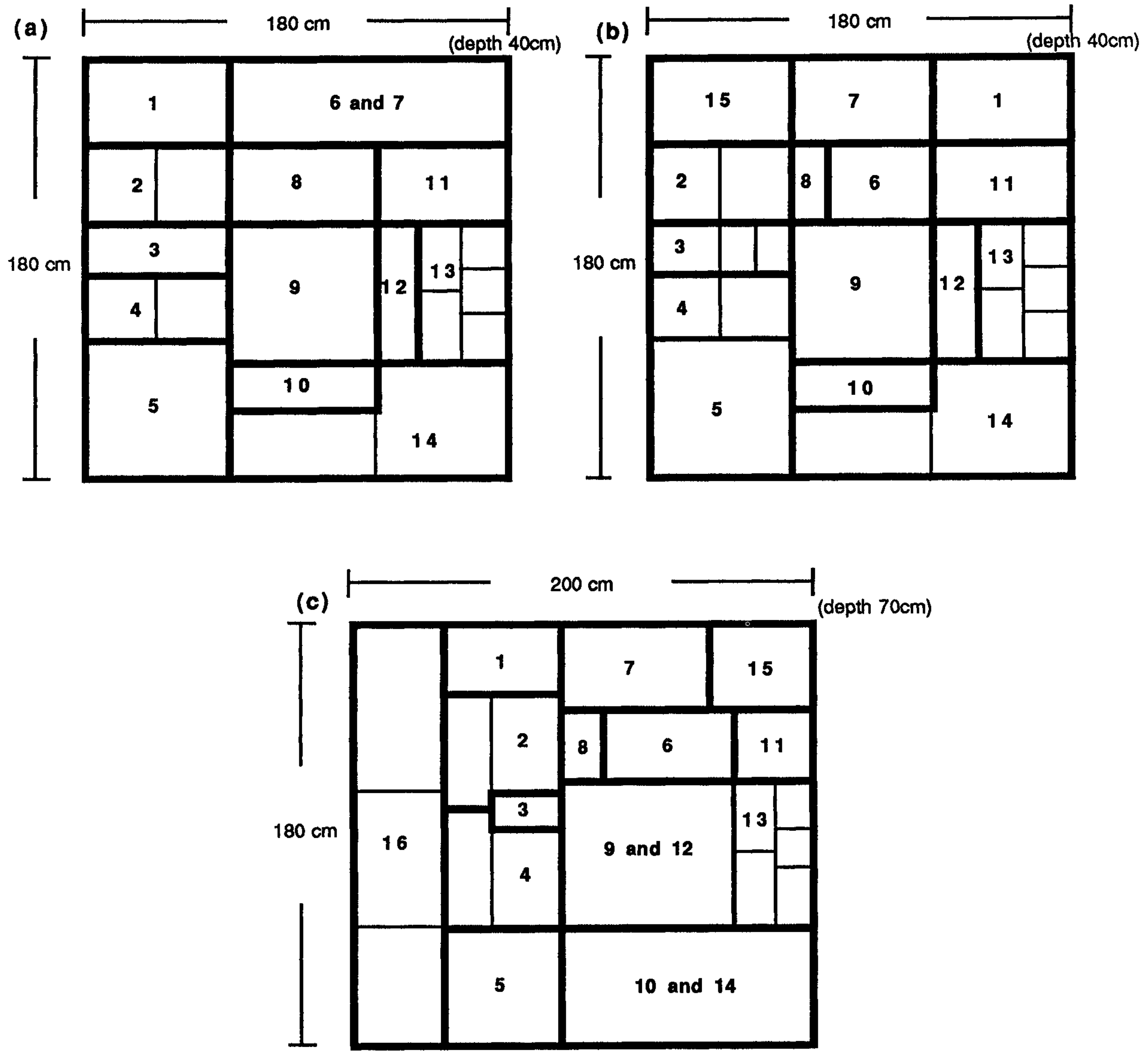

*Figure 3. General layouts of automated synthesis systems—(a) MAVIS; (b) MATES; and (c) EASY.*

1. Switch panel; 2. detector for monitoring HPLC; 3. HPLC injection unit; 4. detector for preparative HPLC; 5. fraction collector; 6. reagent reservoirs; 7. solvent reservoirs; 8. volumetric unit; 9. reaction unit; 10. circulation unit; 11. washing solvent reservoir unit; 12. pH adjustment unit; 13. extraction/separation and drying unit; 14. temperature controlling unit; 15. storage space; 16. control unit (computer, interface etc.). Control unit is set apart for type (a) and (b).

immisicible organic and aqueous phases means that their interface can be detected, and they can be separated. The separation/extraction funnel (SF) contains 10 platinum electrodes which check the conductivity at all points. When the figure is stable at any height, the layers are separated and when a difference is detected at the bottom pair, the separation is stopped. An extraction or washing procedure can be easily modified according to the user's demands.

*Purification and monitoring units*

Figure 7 shows the purification and monitoring units. The preparative HPLC device has two columns, an HPLC pump (SSC-100 K, Senshuu Kagaku, Japan), a UV-detector (ERC-7211, Erma Inc., Japan), 15 solenoid valves and a six-way rotary valve (E1E-012, Uniflows Co., Ltd) fitted with a G–L sensor. The syringe (TP), attached at the end of the 30 ml loop, pulls slowly and stepwise in order to introduce the solution in the solution reservoir (SR3) to the loop. The G–L sensor detects the beginning and the completion of the flow of fluid from SR3 to the loop, and controls the rotary valve switching to inject all of the solution to the column. The fraction collector, designed especially for the automated synthesis system, has 30 receivers (100 ml) and a nozzle. The nozzle can be moved up and down, so that the desired fractions can be transferred back into any of the reaction flasks for further reaction.

The monitoring unit includes a column, an HPLC pump (LC-6A, Shimadzu, Japan), a UV detector (SPD-6A, Shimadzu), 10 solenoid valves, a six-way rotary valve, and two G–L sensors. An aliquot of a reaction solution

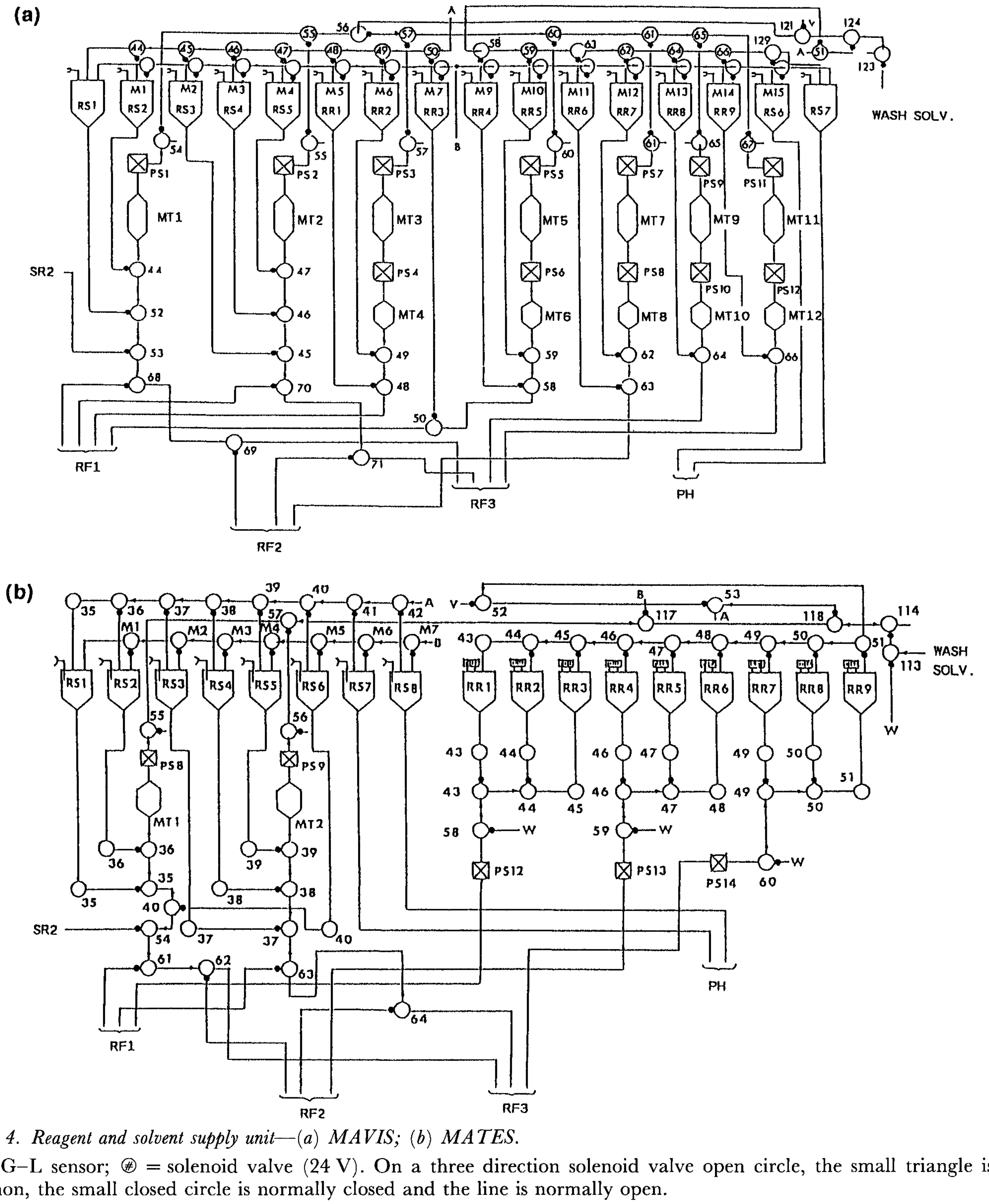

*Figure 4. Reagent and solvent supply unit—(a) MAVIS; (b) MATES.*

PS = G–L sensor; ⊛ = solenoid valve (24 V). On a three direction solenoid valve open circle, the small triangle is common, the small closed circle is normally closed and the line is normally open.

(0·5 ml) is sucked from any of the reaction flasks (RF1, RF2 or RF3). It is then diluted to 10 ml with an appropriate solvent stocked in the corresponding RS, stored in SR2, and injected into the analytical HPLC in order to monitor the reaction.

HPLC data and the chromatogram of both preparative and monitoring procedures are printed out and saved on a floppy disk.

*Additional service units*

Figure 8 shows the flow lines of the temperature control unit and the washing–exhaust/drainage unit. The temperature control unit consists of two circulation pumps, 12 solenoid valves and two baths containing hot and cold ethylene glycol.

The flow lines, including supply units, are washed and

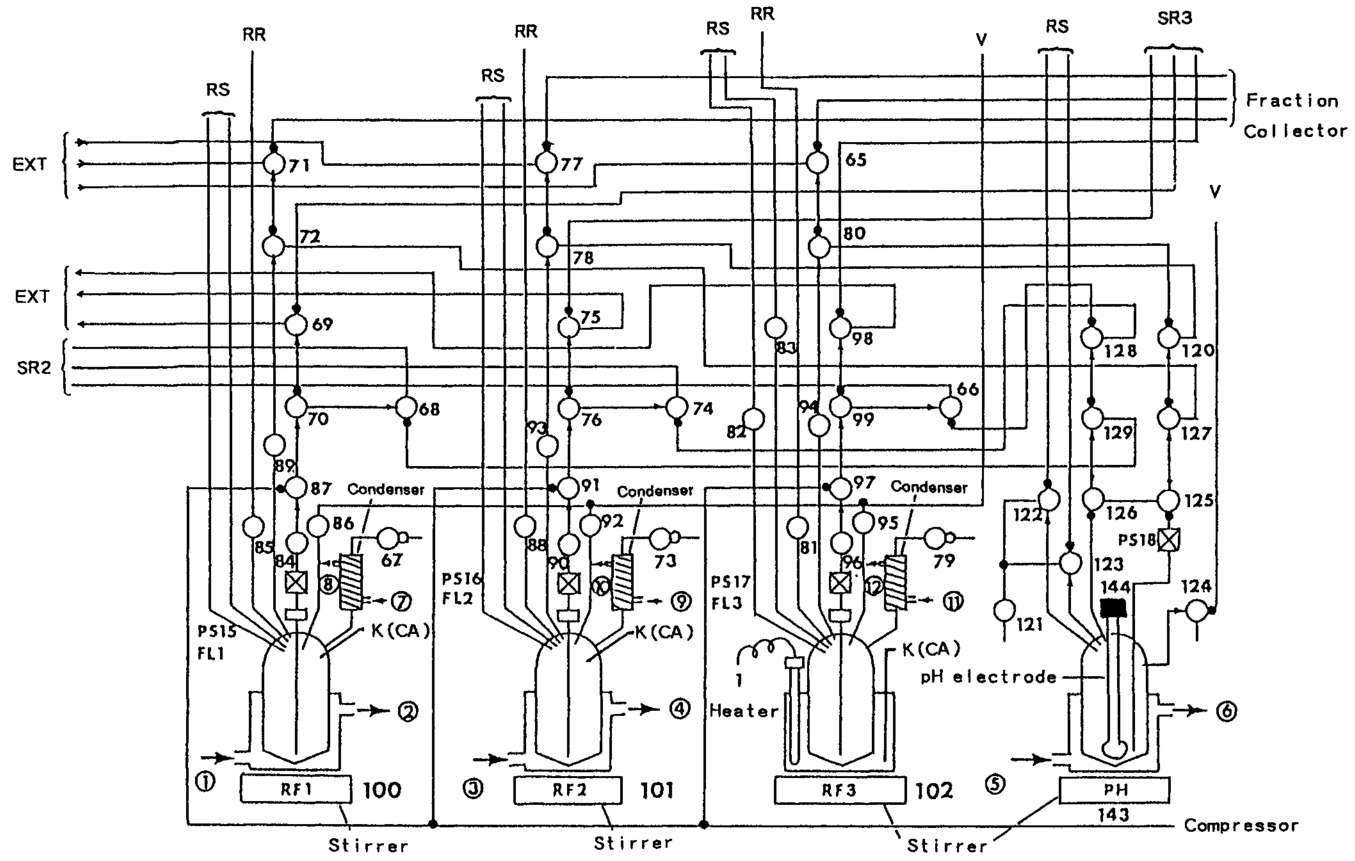

*Figure 5. Reaction unit and pH adjustment unit.*
→ = Inlet and outlet of heating/cooling fluid, ⊕ = solenoid valve (24 V).

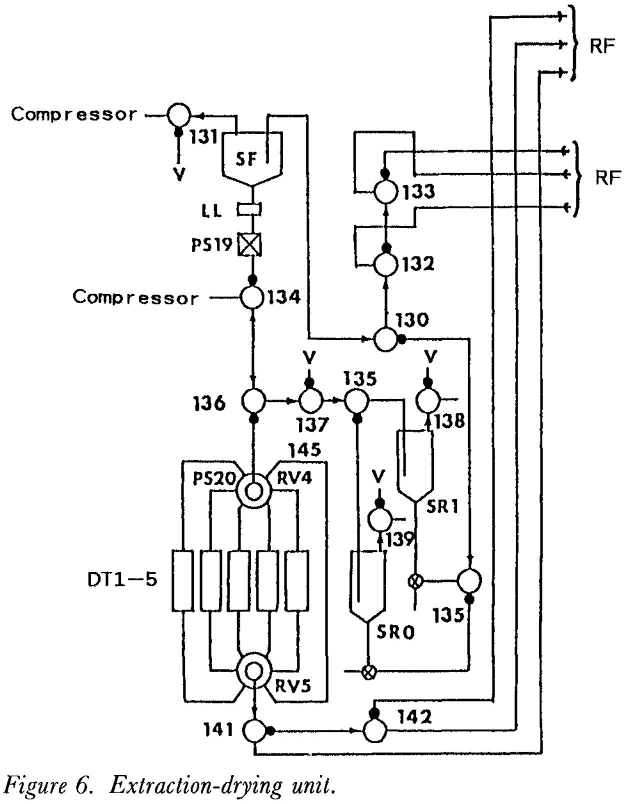

*Figure 6. Extraction-drying unit.*
⊕ = solenoid valve (24 V); RV = rotary valve; DT = drying tube; ⊗ = manual three-way valve.

dried after each run. The ventilation lines of the apparatus are connected to an exhaust duct and the vapour is condensed by a cold trap so that it does not escape into the atmosphere.

### Computer control system

The construction of the computer system and interfaces is shown in figure 9. An NEC PC-9801 series (16 or 32 bit CPU, with an 80–120 MB hard disk) was used as the control system. An I/O expansion unit (PC-9811L, NEC, Japan), consisting of an Intelligent AD-converter (AD12-16S(98)H, CONTEC Co. Ltd, Japan), an input/output board (PIO-24/24(98), CONTEC) and three output boards (PO-48(98), CONTEC), is connected to the main CPU. This expansion unit connects each interface board to the synthesis apparatus in order to register signals from the detectors and sensors via input lines, and to operate the various solenoid valves and relays via output lines. The AD12-16S(98) input board receives signals from the L–L sensor, the UV detectors, the pH meter and the concentration sensors. The input/output board receives signals from 20 photosensors, and the output boards send signals to 160 switches of the synthesis apparatus. An RS232C port is used for the exchange of signals between the CPU and the apparatus in order to control the temperature of the reaction flasks.

The apparatus includes an auto/manual changeover

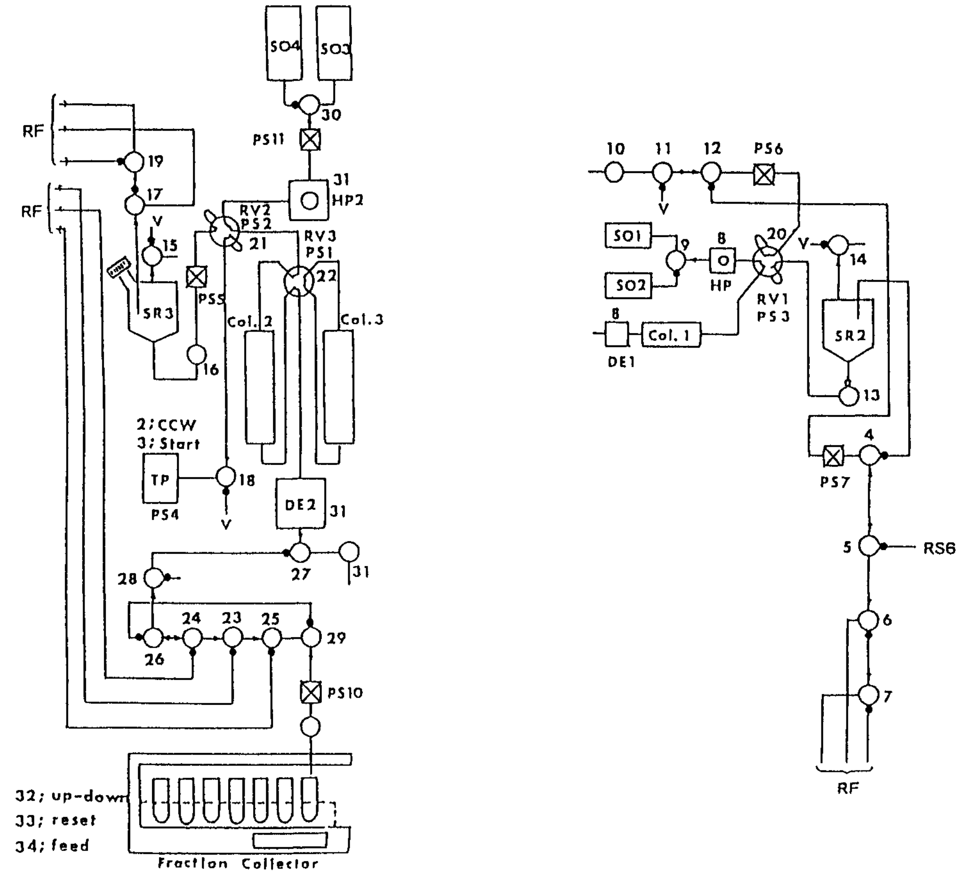

*Figure 7. Purification and monitoring units.*

*SO* = solvent reservoir; PS = G–L sensor; DE = UV-detector; Col = separation column; HP = high pressure pump; RV = rotary valve; ⊕ = solenoid valve (24 V).

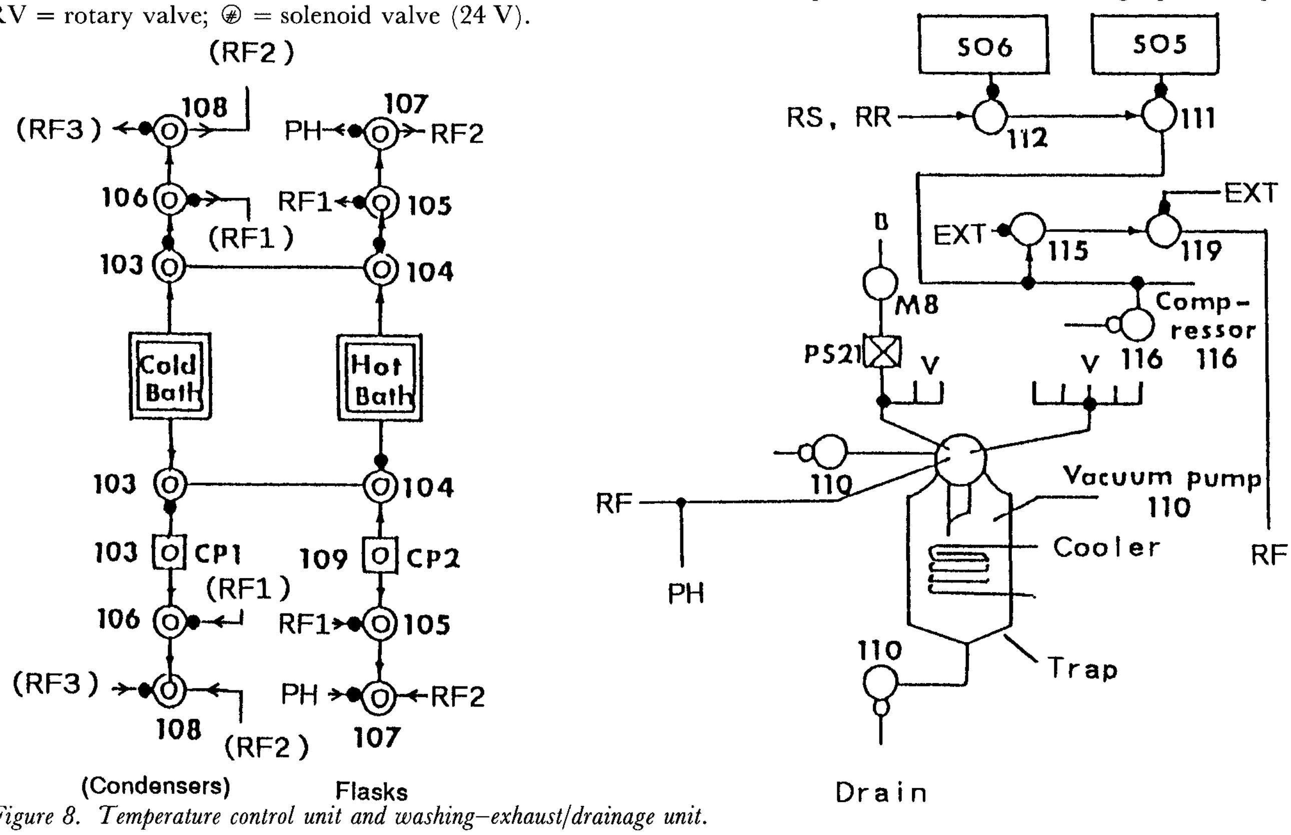

*Figure 8. Temperature control unit and washing–exhaust/drainage unit.*

WS = washing solvent reservoir; CP = circulation pump; → = inlet and outlet of heating/cooling fluid; PS = G–L sensors; ⊕ = solenoid valves (24 V); ◎ = solenoid valves (100 V).

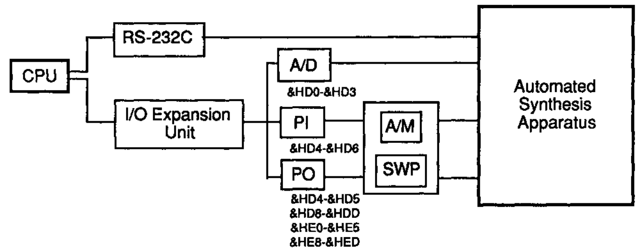

*Figure 9. Interfaces between the automated synthesis system and the computer.*

A/M = auto/manual changeover switch; SWP = switch panel.

*Table 2. Reaction subroutine pools in VACOS, MATES, TARO and EASY.*

| Title | Function |
|---|---|
| START | First subroutine to input all required parameters |
| $RS_x$–$RF_y$ | Measure vol. of liquid in $RS_x$ in measuring tube, transport to $RF_y$ ($x$ = 1–6, $y$ = 1–3) |
| $RR_x$–$RF_y$ | Transport liquid in $RR_x$ to $RF_y$ ($x$ = 1–3, $y$ = 1; x = 4–6, $y$ = 2; $x$ = 7–9, $y$ = 3) |
| $RF_x$–$RF_y$ | Transport liquid in $RF_x$ to $RF_y$($x$ = 1, $y$ = 2, 3; $x$ = 2, $y$ = 1, 3; $x$ = 3, $y$ = 1, 2) |
| $RF_x$–PH | Transport liquid in $RF_x$ to PH ($x$ = 1–3) |
| PH–$RF_x$ | Transport liquid in PH to $RF_x$ ($x$ = 1, 3) |
| $RF_x$–$SR_y$ | Transport liquid in $RF_x$ to $SR_y$ ($x$ = 1, 3, $y$ = 2, 3) |
| $RF_x$–SF | Transport liquid in $RF_x$ to SF ($x$ = 1–3) |
| SF–$RF_x$ | Transport liquid in SF to $RF_x$ ($x$ = 1–3) |
| SF–$SR_x$ | Transport liquid in SF to $SR_x$ ($x$ = 0, 1) |
| $SR_x$–SF | Transport liquid in $SR_x$ to SF ($x$ = 0, 1) |
| SF–$F_x$–$F_y$ | Transport liquid in SF by half to $RF_x$ and $RF_y$ ($x$,$y$ = combination of two out of 1, 2 and 3) |
| FRACT–$RF_x$ | Transport liquid in fraction tubes to $RF_x$ ($x$ = 1–3) |
| $RF_x$–MIX | Stir vigorously ($x$ = 1–3) |
| $RF_x$–STR–ON | Start stirring in $RF_x$ ($x$ = 1–3) |
| $RF_x$–STR–OF | Stop stirring in $RF_x$ ($x$ = 1–3) |
| $RF_x$–BUBB | Bubble in $RF_x$ ($x$ = 1–3) |
| SF–BUBB | Bubble in SF |
| PH–BUBB | Bubble in PH |
| $RF_x$–REA–$n$ | The Nth reaction in $RF_x$ ($\mathcal{N}$ = 1–3, $x$ = 1–3): monitoring available |
| $RF_x$–CON–$n$ | The Nth concentration in $RF_x$ ($\mathcal{N}$ = 1–3, $x$ = 1–3) |
| $EXT_n$ | The Nth extraction ($\mathcal{N}$ = 1–10) |
| $RF_x$–CL–ON | Start cooling $RF_x$ ($x$ = 1, 2) |
| $RF_x$–CL–OF | Stop cooling $RF_x$ ($x$ = 1, 2) |
| $RF_x$–HT–ON | Start heating $RF_x$ ($x$ = 1–3) |
| $RF_x$–HT–OF | Stop heating $RF_x$ ($x$ = 1–3) |
| A–LC–ON | Switch on monitoring HPLC system |
| A–LC–OF | Switch off monitoring HPLC system |
| DE–CO–ON | Switch on preparative HPLC system |
| DE–CO–OF | Switch off preparative HPLC system |
| HPLC | Inject solution in SR3 to HPLC, carry out column chromatography |
| PH–ADJ | Adjust pH of liquid in PH |
| MATU | Wait for 5 min |
| MATU15 | Wait for 15 min |
| ALARM | Alarm and pause until user's interference |
| WASH | Clear screen for washing |
| MR–WASH | Wash MT1, MT2 and RR1–RR9. Washing solvent remains in RF1, RF2 and RF3 |
| WS–$RF_x$ | Transport washing solvent to $RF_x$ via MT1 |
| SF–DR | Drain liquid in SF |
| $SR_x$–DR | Drain liquid in $SR_x$ ($x$ = 0–3) |
| $RF_x$–DRY | Dry $RF_x$ ($x$ = 1–3) |
| RF–DRY | Dry RF1, RF2 and RF3 |
| R–WASH | Wash specified RS |

switch and a switch panel with 160 display switches. The display switch lights are turned on and off corresponding to the valve switches. When the changeover switch is on manual, the user can operate the apparatus by pressing the appropriate switches on the switch panel. Other independent switches for sensors and HPLC systems are also available.

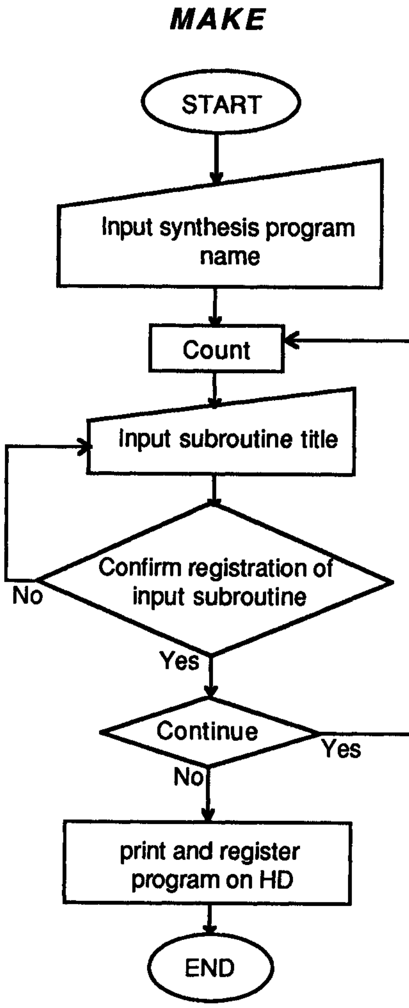

*Figure 10. Flowcharts of the programs available (see text for explanation). (a) Reaction composition program. HD = hard disk (continued over)*

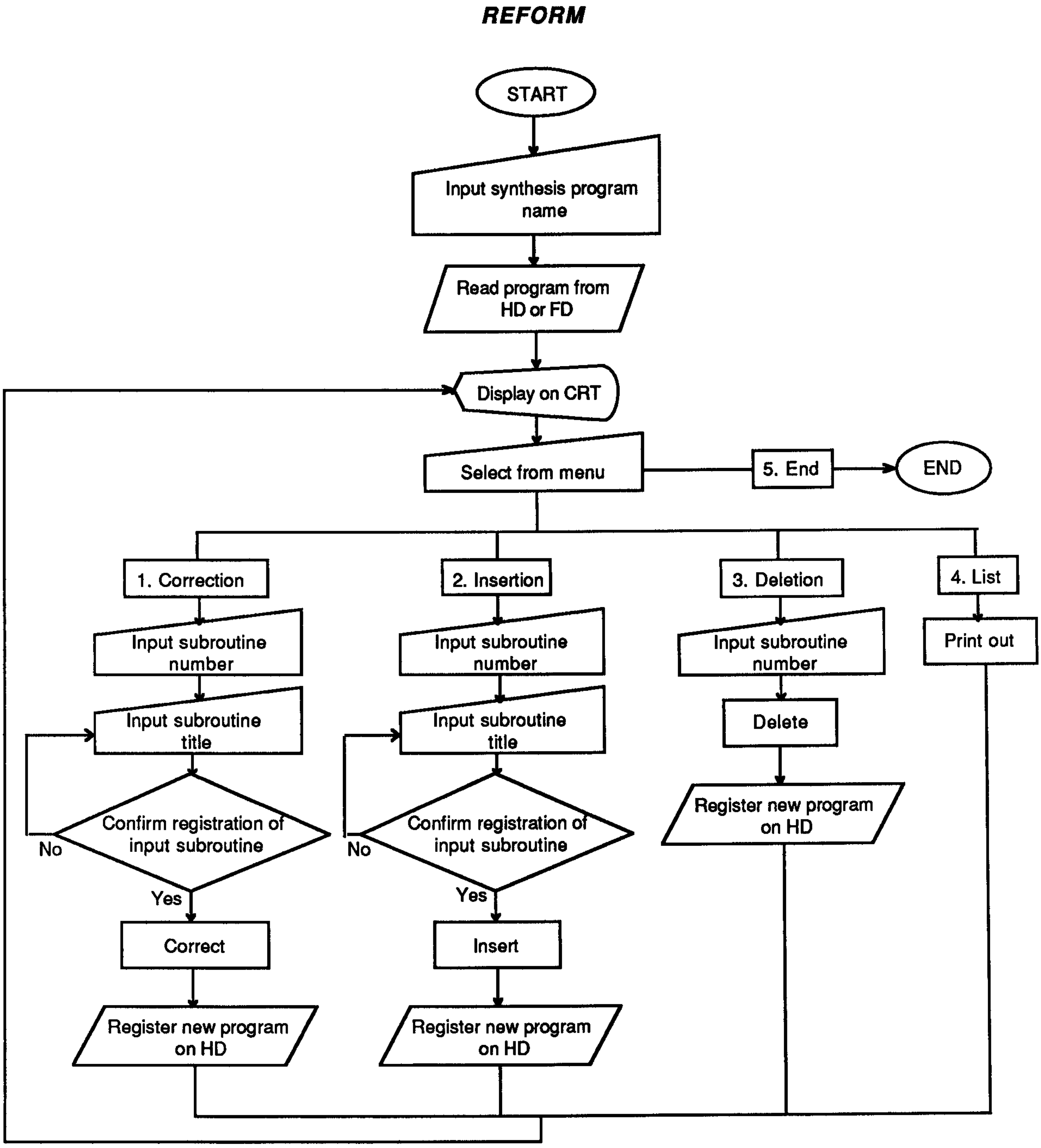

*Figure 10 (continued) (b). Edit program. HD = hard disk; FD = floppy disk. (continued)*

*Software*

The computer runs under MS-DOS and the program is written in N88-BASIC. The user can 'compose' a reaction program easily by drawing one by one from the reaction subroutine pool which contains about 120 subroutines (see table 2). For the reaction work-up procedure, the user can modify or create an extraction module by choosing from the extraction subroutine pool, which has about 60 subroutines (see table 3).

There are five main programs (MAKE, REFORM, SIMU, RUN and CHART) comprising about 25 000 lines in total (see figure 10). The programs are led by menu screens, so they are user-friendly.

The user composes a reaction program by using MAKE, arranges with REFORM, and simulates with SIMU. RUN is used to run the apparatus. When 'START' is chosen as the first subroutine, the parameters for the run can be input (volume, temperature, time, pH, HPLC conditions etc.); the parameters can be saved on a floppy disk and used for the next run. START has a self-diagnosis function—it defines the initial state of the apparatus by checking the sensors and warns the user if any of them is in a wrong position. During operation, the user can freely interrupt the procedure or renew the parameters, either from function keys or from manual operation units.

The program CHART can be used to recall and revise the HPLC data.

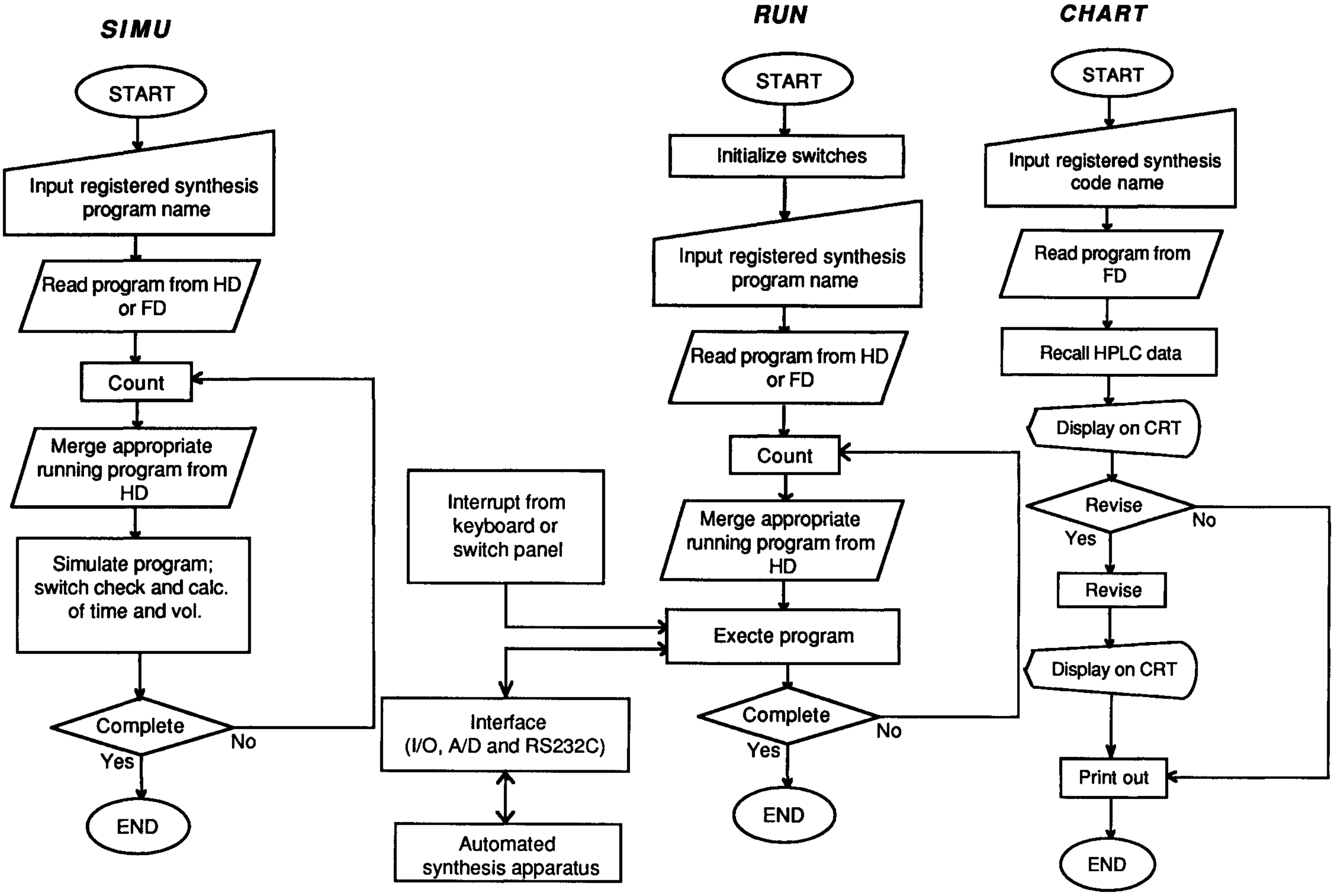

10(*c*) 10(*d*) 10(*e*)

*Figure 10 (continued) (c). Reaction simulation program. (d) Synthesis running program. (e) HPLC data revision program. HD = hard disk; FD = floppy disk.*

*Table 3. Extraction module subroutine pools in VACOS, MATES, TARO and EASY.*

| Title | Function |
|---|---|
| EX-START | Must as the first subroutine |
| EXSLCT | Select extracting solvent, measure, transport to RF |
| EWSSLC | Select washing solvent, measure, transport to RF |
| $ERS_x$–$RF_y$ | Measure volume of liquid in $RS_x$ in measuring tube, transport to $RF_y$ ($x$ = 1–6, $y$ = 1–3) |
| $ERR_x$–$RF_y$ | Transport liquid in $RR_x$ to $RF_y$ ($x$ = 1–3, $y$ = 1; $x$ = 4–6, $y$ = 2; $x$ = 7–9, $y$ = 3) |
| $ERF_x$–SF | Transport liquid in $RF_x$ to SF ($x$ = 1–3) |
| ESF–$RF_x$ | Transport liquid in SF to $RF_x$ ($x$ = 1–3) |
| ESF–$SR_x$ | Transport liquid in SF to $SR_x$ ($x$ = 0, 1) |
| $ESR_x$–SF | Transport liquid in $SR_x$ to SF ($x$ = 0, 1) |
| $ERF_x$–BUBB | Bubble in $RF_x$ ($x$ = 1–3) |
| ESF–BUBB | Bubble in SF |
| $ERF_x$–MIX | Stir vigorously ($x$ = 1–3) |
| ESEP–$SR_x$ | Separate, transport bottom layer to $SR_x$ ($x$ = 0, 1) |
| ESF–DT–$F_x$ | Transport through drying tube to $RF_x$ ($x$ = 1–3) |
| ESF–DR | Drain liquid in SF |
| $ESR_x$–DR | Drain liquid in $SR_x$ ($x$ × = 0, 1) |
| EDT–SLCT | Select drying tube |
| EDT–RST | Reset drying tube |

### Conclusions

The automated synthesis systems described are capable of running continuously 24 hours a day preparing various kinds of organic compounds. They can perform most of the common organic reactions used in the pharmaceutical industry that do not require the use of high pressures or the automated handling of solids. The menu system used for operation means that it is easy for users to compose or modify a program and to operate the synthesizing systems.

Details of the synthesis projects performed with these systems will be published in future papers.

### References

1. Hayashi, N. and Sugawara, T., *Chemistry Letters* (1988), 1613.

2. Hayashi, N. and Sugawara, T., *Tetrahedron Computer Methodology*, **1**(3) (1988), 237.

3. Hayashi, N., Sugawara, T., Shintani, M. and Kato, S., *Journal of Automatic Chemistry*, **11** (1989), 212.

4. Hayashi, N., Sugawara, T. and Kato, S., *Journal of Automatic Chemistry*, **13** (1991), 187.

